# Effects of a Small Increase in Carbon Dioxide Pressure during Fermentation on Wine Aroma

**DOI:** 10.3390/foods9101496

**Published:** 2020-10-19

**Authors:** Lorenzo Guerrini, Piernicola Masella, Giulia Angeloni, Andrea Sacconi, Luca Calamai, Alessandro Parenti

**Affiliations:** Dipartimento di Scienze e Tecnologie Agrarie, Alimentari, Ambientali e Forestali, Università degli Studi di Firenze, Piazzale delle Cascine 16, 50144 Firenze, Italy; piernicola.masella@unifi.it (P.M.); giulia.angeloni@unifi.it (G.A.); andrea.sacconi@stud.unifi.it (A.S.); luca.calamai@unifi.it (L.C.); alessandro.parenti@unifi.it (A.P.)

**Keywords:** aroma modulation, esters, fermentation tanks, Sangiovese wines, sensory analysis

## Abstract

The present study tested the effect of a slight increase in pressure (from 0 to 1 bar) during the fermentation on the wine aroma profile. Fermentations were carried out with a commercial dry yeast on Sangiovese juice in the absence of berry skins. The wine samples fermented under slight overpressure conditions were found to be significantly different from the control samples produced at atmospheric pressure in relation to several chemical compounds. Concentrations of many esters (i.e., isoamyl acetate, ethyl acetate, ethyl hexanoate, hexyl acetate, ethyl dodecanoate, and ethyl tetradecanoate), and acids (i.e., hexanoic acid, octanoic acid, and decanoic acid) increased, while concentrations of two acids (i.e., isobutyric and isovaleric acid) decreased. These differences, notably the higher concentration of esters, are usually associated with a more intense fruity attribute. Triangular sensory tests revealed that the significant chemical differences were also perceivable; hence, introducing a slight pressure increase during the alcoholic fermentation could be a useful tool in managing the aroma profile of wine.

## 1. Introduction

Aroma improvement and modulation are important concerns in the winemaking process for which oenology offers a wide range of applicable methodologies and technologies. Microbial modulation [1], grape post-harvest degradation [2], temperature control during fermentation [1,3], and stem contact fermentation [4,5,6] can all be considered examples of traditional practices that aim to change wine flavor. More recently, other techniques have been developed, such as the recovery of aroma losses during fermentation [7,8] or grape ozone treatment [9]. These methodologies significantly change the wine aroma by modifying the typologies and/or the concentrations of the grape derived aroma compounds, and the microbially derived secondary metabolites [10].

Traditional winemaking techniques that significantly impact wine aroma use different carbon dioxide pressures; examples include carbonic maceration [5,11,12] and second fermentation of sparkling wines [13,14,15]. In the carbonic fermentation, whole berries are maintained in an atmosphere saturated with carbon dioxide for one or two weeks. During this period, small amounts of ethanol are produced [12] and, consequently, pressure in the maceration tank rises slightly. Sparkling wine production employs a similar second fermentation process that can occur either in the bottle, or in a pressurized stainless-steel tank. This step is required to form their characteristic carbon dioxide bubbles. Another alcoholic beverage that is produced with the fermentation at high pressure is beer [16]; a profound change occurs in the volatile profile, with ethyl esters, acids, higher alcohols, and their acetates being the most affected compounds. During the production of both sparkling wine and beer, high carbon dioxide pressure (i.e., higher than 1 bar) significantly reduces the concentration of the above compounds, since pressure affects yeast activity [14,16].

Recently, new technologies that produce a pressure slightly higher than the atmospheric pressure during the alcoholic fermentation have been developed. For example, the Nectar Tank (Trecieffe, Italy) uses carbon dioxide produced by the fermentation to enliven the juice and protect wine from oxidation. Such tanks are called isobaric fermenters. Similarly, the ACDF system (Dynamic Analysis of Fermentation Kinetics; Parsec, Sesto Fiorentino (FI), Italy) applied for fermentation monitoring, uses a pressure switch (set to 0.15–0.30 bar) to monitor fermentation kinetics and to enhance the pressure value. Furthermore, the Ganimede system uses a slight overpressure to achieve the pump-over effect of grape must on skins. For this reason, it could be grouped with the other systems conducting the fermentation at pressure higher than the atmospheric. According to the marketing information available in the respective company’s websites, these systems are able to enhance the extraction from berries. However, the effect of the slightly increased pressure on the aroma of the produced wines has not been studied yet since the studies found in the literature tested higher pressure conditions (higher than 1 bar e.g., sparkling wines and beers). Furthermore, the wine literature is mainly focused on the impact of overpressure conditions after the second fermentations, whereas scant (or no) information is reported on the effect of these pressures after the first fermentation. The hypothesis behind the present study is that a slight carbon dioxide overpressure affects both microbial metabolism and metabolites solubility, resulting in different wine aroma profiles. As carbon dioxide also contributes to extraction from skins, the experiment was conducted on juice only with the aim of separating those 2 effects. Hence, we investigated at the laboratory scale, the effect of a slight increase in pressure (between 0.2 and 1 bar) during the first fermentation of wines on the concentration of the microbially derived secondary metabolites, hence on the volatile profile of the produced wines. Finally, we verified the sensory difference with a discriminant test, using 48 judges, and with quantitation of selected volatile compounds, using head space, solid phase micro extraction, gas chromatography coupled with mass spectrometry (HS-SPME-GC-MS).

## 2. Materials and Methods

### 2.1. Experimental Trials

Five fermentations at different pressure levels were carried out:

- Atmospheric pressure (0 bar, the control sample—the P0 sample),
- Four levels of pressures slightly higher than the atmospheric pressure (0.2, 0.5, 0.7, and 1 bar—the P02, P05, P07, and P1 samples, respectively)

Three replicates were performed at each pressure, and for each of them 2.5 kg of Sangiovese juice were used. The chemical composition of the juice is reported in Table 1. Berry skins were removed from the juice before fermentation to avoid any confounding effects due to an increased concentration of carbon dioxide in the medium [17]. Thus, our study focused mainly on changes in microbially derived secondary metabolites, as grape juice was the same for all treatments, and the wines were analyzed at racking.

The juice was inoculated with 0.4 g/L of commercial dry yeast (ENARTIS Ferm, San Martino (NO), Italy). The must was taken from a unique destemmed and pressed grape juice mass. Three aliquots (i.e., 20 L each) of this must were frozen and used for the 3 replicates. Fermentation was carried out in five, identical, stainless steel sealed pots with a total capacity of 5 L thermostated at 25 °C, and sequentially repeated 3 times. Each pot was equipped with a manometer to monitor the internal pressure, a pressure switch set at one of the five pressure levels, and a tap for sampling. The tap was connected to a flexible pipe submerged in the juice. When the tap was opened, the pressure pushed the juice out, making it possible to collect a sample without opening the pot and changing the internal pressure. Before starting the trial, each pot was tested with a compressor to verify that it was airtight and that each switch opened at the right pressure.

### 2.2. Determination of Volatile Compounds

Headspace solid-phase microextraction coupled with gas chromatography–mass spectrometry (HS-GC-MS) was used for fermentation kinetic monitoring and for volatile compounds determination. The GC used was an Agilent 7820 gas chromatograph, while the MS was a 5977 MSD with electron ionization ion source (Agilent, Santa Clara, CA, USA). A 3-phase (Carboxen/PDMS/DVB) 75 µm–1 cm long fiber (Supelco, Sigma, Darmstadt, Germany) was used.

Fermentation kinetics were controlled by measuring the ethanol content in the samples. Each wine sample was monitored during the alcoholic fermentation: at the beginning of the process (0 days), during the fermentation (after 2, 4, 6 days), and at the end of the fermentation (7 days) for a total of five sampling times. The ethanol range was 0–14%, determined using a 7-point calibration. Methanol D4 was used as the internal standard for ethanol determination. Specifically, 1 mL of wine was added to a 40 mL vial with 10 µL of methanol D4 (Internal Standard—ISTD) and NaCl until oversaturation. Then, 250 µL of air from the sample headspace was injected directly into the column (HP-INNOWax Agilent, 30m × 0.25 mm i.d. × 0.5 µm df mn—Agilent, Santa Clara, CA, USA) with a split ratio of 1:10. The temperature ramp was as follows: after 0.25 min of equilibration, the temperature was maintained at 35 °C for 1 min, then increased at 10 °C/min to 80 °C. Finally, the temperature was increased at a rate of 50 °C/min to 260 °C. The total run time was 14.1 min.

Once fermentation had ended (i.e., day 7), the aroma compounds were determined following the method described by Domizio and co-workers [18,19]. Briefly, the fiber was exposed in the wine headspace for 5 min at 60 °C for volatile compound sampling. Consistent SPME extraction conditions were ensured by a Gerstel MPS2 XL autosampler, equipped with a temperature-controlled agitated tray (Gerstel, Mülheim an der Ruhr, Germany). The column was a HP-INNOWax-Agilent (50 m, 0.2 mm, and i.d. 0.4 µm DF). The injection temperature was 250 °C in splitless mode. Oven temperature started at 40 °C and was maintained for 1 min. The temperature was then increased to 60 °C at 2 °C/min, to 150 °C at 3 °C/min, to 200 °C at 10 °C/min, and finally, to 260 °C at 25 °C/min. The final temperature was held for 6.6 min. In order to normalize the analyte area, the following internal standards were added to samples and calibrations: ethyl acetate D8, butanol D10, ethyl hexanoate D11, acetic acid D3, hexanoic acid D11, 3,4-dimethyl phenol, and 5-methyl hexanol. The complete list of the quantitated compounds, their calibration equations, the R^2^, and the internal standards (ISTD) used for each compound are reported in Table 2.

### 2.3. Sensory Tests

Sensory tests were carried out one week after the fermentation ended (i.e., day 14). From the end of the fermentation to the sensory test, wines were stored in filled containers and the yeasts lees were removed. Two different sensory tests were carried out. A triangle test [21] was performed to identify the perceivable differences between two wine samples: (i) the samples produced at the lowest pressure level (i.e., 0.2 bar—the P02 samples) and (ii) the control samples (i.e., produced at atmospheric pressure—the P0 samples). The choice of analyzing the differences between the above samples was made since these samples showed the highest differences in the volatile compound composition. In order to obtain an amount of wine sufficient for the sensory tests, the three replicates of each pressure were merged together, and the resulting wines were presented to the judges. In detail, three coded samples were presented to panelists: two were identical and one was different. Samples were identified with a random 3 digit code. They were served to judges at room temperature (roughly 20 °C) in standard tasting glassed filled with about 30 mL of wine. The 48 members of the panel were asked to identify the odd sample from its odor. Judges were regular wine consumers, in the range of 20–57 years old. Twenty one of them were females, while 27 were males. The second sensory test performed was a preference test; all five samples produced at the five pressure levels (i.e., the P0, P02, P05, P07, and P1 samples) were presented to the panelists, and the judges were asked to state which one they preferred. Specifically, they had to answer to the following question: “Among the 5 samples, which one do you prefer?” (i.e., in Italian “Fra i 5 campioni quale preferisci?”).

### 2.4. Statistical Analysis

In the first step, a principal component analysis (PCA) was applied to the data to screen the variables. Hence, all the chemical measurements were used as input variables for the PCA. Before performing the PCA, data were scaled and centered. Then, we verified by type III analysis of variance (ANOVA) that no significant differences among replicates occurred. After that, the concentration of each compound was tested with a one-way ANOVA, to assess the differences among pressures. When the ANOVA was significant (*p* < 0.05), a Tukey Honestly Significant Difference (HSD) post hoc test was performed. For sensory trials, the chi-squared test was used for the comparison between expected (random choice of judges) and measured frequencies. The null hypothesis was rejected when *p* < 0.05. R software (version 3.6.0) was used for all data processing. In the R software, the function aov() was used for the ANOVA, the function TukeyHSD() for the post hoc test and the function chisq.test() for the chi-squared test. Tabulated data were used to assess statistically significant differences in triangular test.

## 3. Results and Discussion

The fermentation was monitored every two days. It ended on day six, and a similar trend was observed in all the wine samples. No significant difference was found for ethanol content (mean 12.8 ± 0.2%) for all pressure levels tested and sampling times (days 0, 2, 4, and 6). Hence, pressure did not affect ethanol production. Consistently, at the end of the fermentation, all the samples showed less than 1 g/L of residual sugars. The acidity of the obtained wines was on the average 6.9 ± 0.2 g/L, without showing significant differences among the five pressure levels.

**Figure 1:**
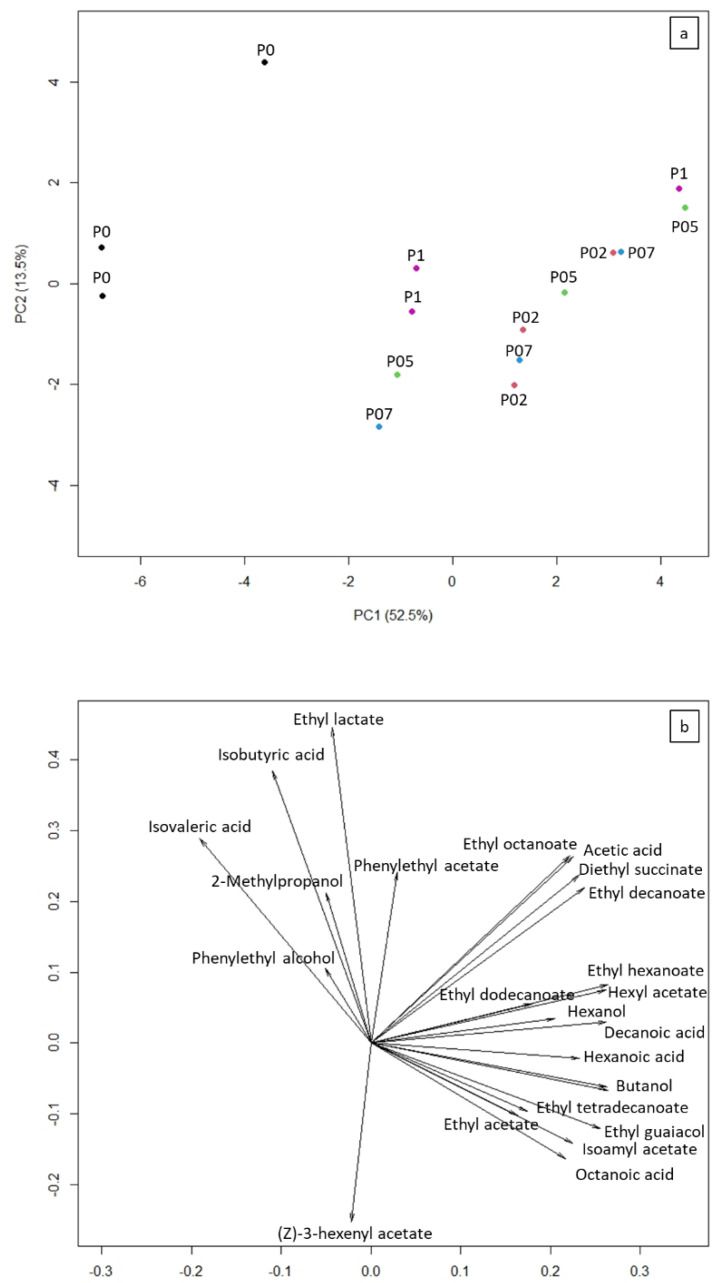
Score (upper graph (**a**)) and loading (lower graph (**b**)) plots obtained with the principal component analysis (PCA). In the score plot, black points represent the control samples produced at atmospheric pressure (i.e., 0 bar—the P0 samples), red points the P02 samples (i.e., 0.2 bar), green points the P05 samples (i.e., 0.5 bar), blue points the P07 samples (i.e., 0.7 bar), and purple points the P1 samples (i.e., 1.0 bar).

Seven days after fermentation ended, two sensory tests were performed, and the 26 compounds reported in Table 3 were quantitatively measured. The PCA clearly separated the control wines fermented at atmospheric pressure from all of the other samples fermented in overpressure conditions. PC1 explained 52.5% of the total variance, while PC2 explained 13.5% (Figure 1). The P0 samples were placed in the left side of the score plot, while the P02, P05, P07, and P1 samples in the right side (Figure 1a). Hence, PCA first loading was of particular interest since it allowed to identify the compounds that produced the aroma differences between atmospheric and overpressure conditions (Figure 1b). The wine samples placed in the right side of the score plot were characterized by higher concentrations of compounds with positive loadings and lower concentrations of compounds with negative loadings. The opposite result was obtained for the wines placed in the left side of the plot. The highest loadings were found for esters (i.e., ethyl acetate, isoamyl acetate, butanol-3-methyl acetate, hexyl acetate, ethyl hexanoate, ethyl octanoate, ethyl decanoate, etc.), alcohols (i.e., 1-buanol, 1-hexanol), and some acids (i.e., acetic, octanoic and decanoic acid). Thus, the above chemical compounds can be considered as the main contributors to the aroma difference between the control samples and the samples produced in overpressure conditions. On the other hand, the highest negative scores were found for isobutyric and isovaleric acid, which were found to be higher in the P0 samples.

The ANOVA highlighted significant (*p* < 0.05) differences for 13 compounds making up the aroma profile (Table 3). Seven of the above compounds belong to the ester class (i.e., ethyl acetate, ethyl hexanoate, hexyl acetate, isoamyl acetate, ethyl dodecanoate, ethyl tetradecanoate, and phenylethyl acetate), five to the acid class (i.e., isobutyric acid, isovaleric acid, hexanoic acid, octanoic acid, and decanoic acid), and one to the alcohol class (i.e., 1-butanol).

Esters are usually related to the fruity flavor [20] and, in general, they were found to increase in the wine samples produced under overpressure conditions compared to the control samples [21]. Ethyl acetate is a well-studied volatile compound. In our trials, ethyl acetate concentration in the *P*0 samples remained under the odor threshold (OT), while in the P02, P05, P07, and P1 samples ethyl acetate concentrations were significantly higher. Thus, the overpressure treatment, regardless the pressure level, made the ethyl acetate perceivable in the obtained wine samples. Some authors considered ethyl acetate a positive compound if its concentration remains below 200 mg/L [22], as in our trials. However, ethyl acetate could have a suppressive effect on the perception of other esters and, consequently, it could negatively affect the wine flavor [20].

The same result was obtained for ethyl hexanoate. In the literature, this compound is associated to fruity notes, in particular to a red berry aroma associated with “strawberry jam/red berry fruit/raspberry jam” [23], and can be considered a positive compound for the aroma of fermented beverages [24]. Similar to ethyl acetate, concentrations of ethyl hexanoate were significantly lower in the control wine samples compared to the samples fermented at higher pressures.

**Figure 2:**
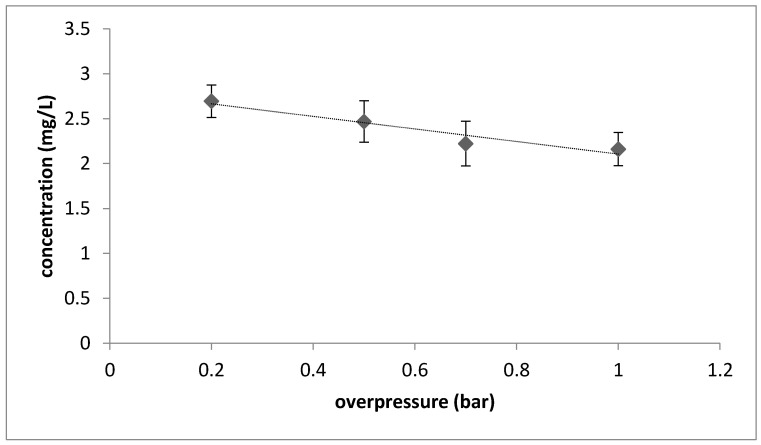
Decrease in isoamyl acetate concentration (mg/L) as a function of the level of overpressure (from 0.2 to 1 bar) applied during the fermentation. Error bars represent the standard deviation; the trend line was calculated with a linear model (R^2^ = 0.76).

Isoamyl acetate concentrations were around 5.6 times higher in the P1 samples compared to the P0 samples. This compound is described as “sweet, fruity, banana, solvent” in wines [14]. Thus, conducting a fermentation in overpressure significantly impacted the concentration of this ester. A regression (removing the P0 samples) to test for a decrease in isoamyl acetate related to overpressure increase was performed. Results showed that, for almost all of the aforementioned compounds, the highest concentrations in volatile compounds were obtained at the lowest pressure level (i.e., 0.2 bar), and the lowest concentrations at the highest-pressure level (i.e., 1 bar). An example is the significant decrease in the isoamyl acetate concentration as pressure increased (*p* = 0.02). The highest isoamyl acetate concentration was produced when the lowest level of overpressure was applied; further pressure increases significantly reduced the concentration of this compound (Figure 2). The literature reports contrasting results regarding the isoamyl acetate produced during fermentation in overpressure conditions. Martínez-García et al. [14] reported a significant decrease of isoamyl acetate concentration during the second fermentation of a sparkling wine. Similarly, Renger et al. [16] reported a reduction of the concentration of this compound during beer fermentation in overpressure (in the range 1–3 bar). Conversely, Tesniere and Flanzy [12] showed a significant increase of isoamyl acetate concentration during the carbonic maceration (at slight overpressure) for the production of a Beaujolais wine. A possible explanation of the obtained results could be that a slight overpressure condition is responsible for the increase of the isoamyl acetate concentration in wines, by increasing the compound solubility, whereas further increases in pressure cause a significant reduction of this compound. Indeed, the wine samples produced at moderate overpressure conditions showed a higher concentration of this ester, while the wine samples obtained at higher pressure conditions were characterized by lower concentrations. Similar to isoamyl acetate, a significant linear decrease (*p* = 0.04) was found for ethyl hexanoate concentrations as function of overpressure level. Finally, ethyl acetate reached the highest concentration at the lowest overpressure level (0.2 bar), and, like isoamyl acetate and ethyl hexanoate, its concentration significantly decreased when increasing the pressure level.

An explanation for the decrease in ester concentrations with increasing the overpressure level can be found in the beer and wine literature. A decrease in ethyl acetate when fermentation occurs at a pressure of 1 bar or more is reported in literature [25,26]. Ethyl acetate biosynthesis is mediated by acetyl-CoA activity, which, at pressures above 1 bar (i.e., in the latter studies) is inhibited by carbon dioxide [27]. This phenomenon does not occur at lower pressure levels (i.e., the pressure levels tested in the present study) where other phenomena are responsible for the significant increase of its concentration. Furthermore, Tesniere and Flanzy reported an increase in ethyl acetate concentration during carbonic maceration (at slight pressure) in the production of a Beaujolais wine [12], while Martínez-García et al. [14] reported a decrease during the second fermentation of a sparkling wine at pressure higher than 1 bar. Similarly, a reduction during the fermentation of beer produced under pressure conditions (in the range 1–3 bar) is reported in literature [16]. These findings could reveal that a slight increase in pressure is able to increase isoamyl acetate concentrations in wines, while pressure higher than 1 bar may decrease it.

Hexyl acetate is another pleasant compound, related to the red berry aroma [28]. Similar to the results obtained for ethyl acetate and ethyl hexanoate, hexyl acetate concentrations were found to be significantly higher in the wine samples produced under overpressure conditions compared to the control samples. In all the wine samples produced with overpressure the concentrations were over the OT (1.50 mg/L). Their odor activity values (OAV) ranged from 1.9 to 2.4, whereas the control samples showed a value lower than the OT threshold (OAV = 0.5). This compound is related to the carbonic maceration of grapes [11] and has been found to decrease during the second fermentation of sparkling wines [14].

Other esters showing higher concentrations in the samples produced under overpressure conditions were ethyl dodecanoate and ethyl tetradecanoate. The former is known to be a contributor to the fruity aroma [29] and has been found in sparkling wines [14], carbonic maceration wines [11] and beers [24]. The latter is described as fruity, like the aforementioned esters, but also as “butter, fatty” [14]. Hence, ethyl tetradecanoate can be considered as the least-desirable ester among those detected. However, since both compounds were found to be below their respective OT (0.8 mg/L and 2.0 mg/L) in all the analyzed samples, they had very little impact on the overall taste of the resultant wines. Their average OAV were 0.5 for ethyl dodecanoate and 0.1 for ethyl tetradecanoate.

Phenylethyl acetate differed from the other esters since the lowest concentration was found in the P07 samples (i.e., 0.7 bar). This compound is described as “rose, honey, tobacco” [20]. In the literature, it has been related to the second fermentation of sparkling wines [14], and has been found to decrease during storage in contact with lees [29].

To summarize, slightly increased pressures applied during fermentation significantly increased the concentration of the compounds related to fruity aromas. This result is consistent with the literature reporting that ethyl acetate, isoamyl acetate, and ethyl hexanoate are the main contributors of the fruity flavor in wine [7,20].

Overpressure conditions applied during fermentation significantly changed the acid profile of the wine samples. Isobutyric, isovaleric, hexanoic, octanoic, and decanoic acids were found to be significantly different. Concentrations of isobutyric and isovaleric acids showed a significant decrease as pressure increased. Both of the above acids are undesirable compounds in wines. They are described as “rancid, butter, cheese” (isobutyric), and as “sweat, acid, rancid” (isovaleric) [20]. Concentrations of both compounds were found to be the highest in the control wine samples; furthermore, it was found that the concentration of both these acids decreased as pressure increased. Isovaleric acid was perceivable in all the wine samples (i.e., OAV > 1), while isobutyric acid was only perceivable in the P0 samples (OAV = 1.1).

Similar to esters, concentrations of octanoic and decanoic acids significantly grew as pressure increased. However, unlike esters, these compounds can be considered undesirable in wine since both of them are related to a fatty and rancid aroma [20]. Similar changes have been found in carbonic maceration wines (see [11] for a discussion of octanoic acid), while a decrease has been found during the second fermentation of sparkling wines [14]. Nevertheless, in our study, OAVs of these compounds remained lower than 1 in all the wine samples. Finally, concentrations of hexanoic acid significantly increased as pressure was enhanced; in all cases, concentrations were above the OT (OAV > 1), hence perceivable in all the wine samples.

The concentration of one alcohol (1-butanol) significantly increased as increasing the fermentation pressure. The difference was significant after seven days of fermentation. The literature reports that 1-butanol increases during carbonic maceration [11] despite no particular effect on the wine aroma profile was reported for this compound.

The above discussed differences prove that a slight pressure increase during the alcoholic fermentation can change the volatile composition of a wine from a chemical perspective. However, from volatile compositional data, it is almost impossible to understand if pressure caused a sensory change of the wines at racking. Moreover, not all the active compounds for the wine aroma were quantified, and it is very difficult to consider all the interactions among volatiles (i.e., enhancement and masking effects). For these reasons we performed a discriminant sensory test. With the triangular test, we do not want to decide if a wine made with overpressure is better or worse than one made at atmospheric pressure. We only want to prove that the chemical differences due to the overpressure are perceivable by tasters, and consequently, that overpressure fermentation could be a low-cost strategy to modulate wine aroma.

**Figure 3:**
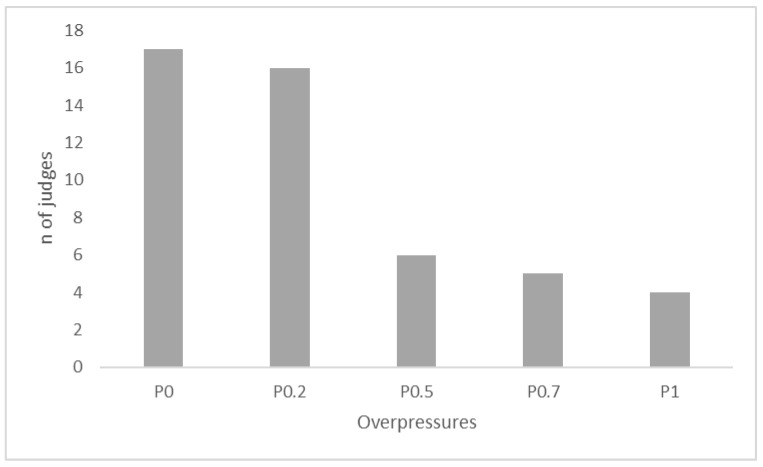
Frequency distribution of preferences as a function of fermentation pressure.

The results of the triangle test were statistically significant (*p* < 0.001): 32 of the 48 judges were able to distinguish the samples produced under pressure conditions from those fermented at atmospheric pressure. This finding was consistent with results of the chemical analyses. The significant differences in concentrations of the volatile compounds identified by GC-MS allowed panelists to discriminate between wine samples obtained in overpressure conditions from the control samples obtained at atmospheric pressure. The results of the preference test are shown in Figure 3.

Approx. one third of judges (i.e., 17) preferred the control samples (0 bar), and approx. one third (i.e., 16) preferred the wine samples produced at the lowest pressure level (0.2 bar). The remaining judges were distributed across the other three wine samples (0.5, 0.7, and 1 bar). Sensory test were consistent with the chemical findings, showing that the greatest difference (in terms of volatile composition) was found between 0.2 bar and 0 bar wine samples. Furthermore, data from GC-MS allowed to split the wine samples in 2 groups: the control wine samples and the wine samples produced in overpressure conditions. In the same way, some judges preferred the control wine samples, whereas some others preferred the increased pressure wines (the wines were different but the preference is individual). Hence, applying pressure during fermentation caused significant chemical differences, which further resulted in wine samples significantly different in their sensory profile (i.e., “odor” attribute) as assessed by judges. Within the wine samples produced at pressures higher than the atmospheric pressure, the samples produced at the lowest overpressure level (0.2 bar) showed the highest concentrations of compounds, measured with GC-MS. This is consistent with the claimed differences in wine aroma profile by the commercial solutions using overpressure during fermentation (which work at low overpressures comparable with the 0.2 bar trial), where we measured the maximum effect on wine.

These results highlighted that a simple and low-cost technological solution was able to significantly change the wine aroma profile.

## 4. Conclusions

The present study showed that a slight increase in pressure during fermentation can significantly change the aroma profile of the produced wines in the absence of berry skin contact. The highest difference was found between the control samples produced at atmospheric pressure and samples obtained at the lowest overpressure level (0.2 bar). Isoamyl acetate was the compound mostly affected by the overpressure treatment. Other esters (i.e., ethyl acetate, ethyl hexanoate, hexyl acetate, and phenylethyl acetate) showed a significant increase with increasing the fermentation pressure, resulting in wine samples characterized by a higher concentration of compounds related with a fruity flavor. The same trend was found for some acids (i.e., hexanoic acid, octanoic acid, and decanoic acid) and for 1-butanol alcohol. Furthermore, two acids related to an unpleasant taste (isobutyric and isovaleric acid) significantly decreased when applying overpressure conditions. The sensory tests confirmed that the differences detected by GC-MS were also well perceivable by judges. However, to understand more regarding the consumer acceptability of wines produced with overpressure during the alcoholic fermentation, further tests are still required. In conclusion, this study showed that the application of a pressure slightly higher than the atmospheric pressure during the alcoholic fermentation can be used to significantly change wine aroma. Further studies testing the effect of this pressure range on both aroma and extraction from berry skins have to be investigated.

## Figures and Tables

**Table 1:** Chemical characterization of Sangiovese grape juice used in the trials.

Parameter	Concentration
Total sugar (g/L)	215
Glucose (g/L)	113
Fructose (g/L)	102
pH	3.37
Titratable acidity (g/L sulfuric acid)	4.43
Free SO_2_ (mg/L)	0
Total SO_2_ (mg/L)	22
Malic acid (g/L)	1.5
Ammonia nitrogen (mg/kg)	56
Amino acid nitrogen (mg/kg)	117

**Table 2:** Compound names, calibration curve, R^2^, and internal standard used for the determination of each volatile compound. List of internal standards (ISTD): (i) ethyl acetate D8; (ii) butanol D10; (iii) o-xylene D10; (iv) ethyl hexanoate D11; (v) butanol D10 acetate D3; (vi) 5-methylhexanol; (vii) acetic acid D3; (viii) naphtalene D8; (ix) hexanoic acid D11; (x) 3,4-dimethyl phenol. OT = odor threshold.

Compound Name	Calibration Equation	R^2^	ISTD	OT (mg/L)	Ref.	Descriptors
Ethyl Acetate	y = 0.014675x + 0.066574	0.99	i	12.26	[7]	pineapple, varnish, balsamic
2-Methylpropanol	y = 0.039772x − 0.003727	0.98	ii	830	[14]	fusel alcohol, ripe fruit
Isoamyl acetate	y = 0.100689	0.92	iii	0.03	[7]	sweet, fruity, banana, solvent
1-butanol	y = 0.089765x − 0.040793	0.99	ii	-	-	-
Ethyl Hexanoate	y = 2.209025x + 0.590842	0.96	iv	0.014	[7]	fruit, fat
*p*-cymene	y = 0.120378x − 0.002342	0.97	iii		[18,19]	citrus, tymus
Hexyl Acetate	y = 0.263802x + 0.008024	0.98	iv	1.5	[14]	green apple
(Z)-3- Hexenyl Acetate	y = 52.46746x + 1.253990	0.98	v	1.9	[18,19]	herbaceous
Ethyl Lactate	y = 0.036195x − 0.029498	0.99	iv	154.7	[7]	
1-hexanol	y = 0.262175x + 0.006559	0.99	vi	8	[7]	grass just cut
Etyhl Octanoate	y = 2.037874x − 0.694484	0.99	iv	0.005	[7]	pineapple, pear, soapy
Acetic Acid	y = 0.049014x − 0.002893	0.99	vii	200	[20]	sour, pungent, vinegar
Isobutyric Acid	y = 0.027054x − 0.006984	0.98	iv	2.3	[20]	rancid, butter, cheese
Ethyl Decanoate	y = 0.020602x + 0.001370	0.99	viii	0.2	[7]	sweet, fruity, nuts and dried fruit
Diethyl Succinate	y = 0.109274x + 0.009381	0.92	viii	200	[7]	overripe melon, lavender
Isovaleric Acid	y = 0.039342x + 0.006740	0.87	iv	0.03	[20]	sweat, acid, rancid
Hexanoic Acid	y = 0.123790x − 0.069588	0.99	ix	0.43	[20]	sweat
2-Phenylethyl Acetate	y = 3.255536x − 1.461896	0.99	ii	0.25	[7]	fruity, rose, sweet, honey
Ethyl Dodecanoate	y = 0.076558x + 0.414303	0.96	iv	1.5	[14]	sweet
Phenylethyl Alcohol	y = 0.767459x − 1.055210	0.99	x	10	[14]	rose talc, honey
b-ionone	y = 2.266933x + 0.034350	0.99	iii	0.45	[18,19]	flowers, violet
Ethyl guaiacol	y = 2.111488x + 0.032071	0.98	x	0.03	[20]	spice, clove
Octanoic Acid	y = 0.590406x + 0.210770	0.97	ix	0.5	[14]	fatty, waxy, rancid, oily
Ethyl Tetradecanoate	y = 0.703345x − 0.001324	0.99	iv	2	[14]	sweet fruit, butter, fatty odor

**Table 3:** Means and standard deviations of quantified aromatic compounds (*p* < 0.05). Concentrations are expressed in mg/L. The presence of different letters indicates statistically significant differences at Tukey honestly significant difference (HSD) post hoc test, while the presence of the same letters means there were no statistically significant differences.

Compound Name	Samples				
	P0 (0.0 Bar)	P02 (0.2 Bar)	P05 (0.5 Bar)	P07 (0.7 Bar)	P1 (1.0 Bar)
*Alcohols*					
1-Butanol	1.77 ± 0.55 ^a^	8.88 ± 1.71 ^b^	9.15 ± 2.82 ^b^	7.95 ± 1.70 ^b^	7.69 ± 1.58 ^b^
2-Methylpropanol	127.37 ± 52.25 ^a^	74.02 ± 32.38 ^a^	53.99 ± 2.95 ^a^	120.13 ± 71.37 ^a^	110.22 ± 44.90 ^a^
1-hexanol	0.62 ± 0.19 ^a^	0.91 ± 0.14 ^a^	0.91 ± 0.06 ^a^	0.92 ± 0.07 ^a^	1.11 ± 0.15 ^a^
Phenylethyl alcohol	85.96 ± 19.08 ^a^	76.76 ± 24.38 ^a^	73.50 ± 18.56 ^a^	69.51 ± 6.98 ^a^	91.01 ± 15.64 ^a^
*Acids*					
Acetic acid	74.03 ± 42.55 ^a^	114.21 ± 34.10 ^a^	119.19 ± 55.04 ^a^	109.07 ± 22.33 ^a^	122.87 ± 34.11 ^a^
Hexanoic acid	0.07 ± 0.12 ^a^	0.96 ± 0.17 ^b^	0.86 ± 0.24 ^b^	0.79 ± 0.48 ^b^	0.85 ± 0.67 ^b^
Octanoic acid	0.36 ± 0.11 ^a^	2.27 ± 0.62 ^b^	2.04 ± 0.45 ^b^	2.08 ± 0.62 ^b^	1.95 ± 0.92 ^b^
Decanoic acid	0.78 ± 0.53 ^a^	3.14 ± 0.47 ^b^	2.95 ± 0.89 ^b^	2.47 ± 1.15 ^b^	2.46 ± 1.10 ^b^
Isobutyric acid	2.55 ± 0.43 ^a^	1.91 ± 0.24 ^b^	1.65 ± 0.29 ^b^	1.23 ± 0.49 ^b^	1.73 ± 0.22 ^b^
Isovaleric acid	2.05 ± 0.35 ^a^	1.01 ± 0.43 ^ab^	0.80 ± 0.17 ^ab^	0.65 ± 0.24 ^c^	0.83 ± 0.09 ^bc^
*Esters*					
Ethyl acetate	6.78 ± 5.61 ^a^	22.42 ± 2.26 ^b^	16.57 ± 0.72 ^b^	21.72 ± 1.93 ^b^	16.66 ± 6.44 ^b^
Ethyl hexanoate	3.15 ± 3.17 ^a^	9.86 ± 1.55 ^b^	10.54 ± 2.76 ^b^	9.01 ± 3.18 ^b^	9.95 ± 1.42 ^b^
Hexyl acetate	0.73 ± 1.11 ^a^	3.39 ± 0.90 ^b^	3.57 ± 1.31 ^b^	2.92 ± 1.04 ^b^	2.96 ± 0.41 ^b^
(Z)-3-hexenyl acetate	0.03 ± 0.01 ^a^	0.05 ± 0.03 ^a^	0.03 ± 0.00 ^a^	0.03 ± 0.02 ^a^	0.03 ± 0.01 ^a^
Ethyl lactate	5.65 ± 2.47 ^a^	3.18 ± 1.23 ^a^	4.48 ± 1.57 ^a^	3.14 ± 0.61 ^a^	5.14 ± 0.91 ^a^
Ethyl octanoate	1.33 ± 0.77 ^a^	2.06 ± 0.68 ^a^	2.16 ± 1.09 ^a^	1.99 ± 0.42 ^a^	2.23 ± 0.66 ^a^
Ethyl decanoate	28.60 ± 20.82 ^a^	66.91 ± 27.35 ^a^	76.89 ± 60.00 ^a^	61.53 ± 28.90 ^a^	70.28 ± 42.93 ^a^
Ethyl dodecanoate	0.37 ± 0.08 ^b^	0.49 ± 0.07 ^a^	0.47 ± 0.08 ^a^	0.42 ± 0.10 ^a^	0.43 ± 0.07 ^a^
Ethyl tetradecanoate	0.12 ± 0.03 ^b^	0.32 ± 0.15 ^a^	0.29 ± 0.05 ^a^	0.22 ± 0.06 ^a^	0.26 ± 0.05 ^a^
Diethyl succinate	0.03 ± 0.03 ^a^	0.07 ± 0.04 ^a^	0.08 ± 0.07 ^a^	0.07 ± 0.04 ^a^	0.08 ± 0.06 ^a^
2-Phenylethyl acetate	3.77 ± 2.77 ^a^	3.48 ± 0.88 ^a^	2.94 ± 1.17 ^a^	2.48 ± 0.93 ^b^	2.93 ± 2.14 ^a^
Ethyl guaiacol	0.08 ± 0.01 ^a^	0.16 ± 0.08 ^a^	0.17 ± 0.08 ^a^	0.22 ± 0.09 ^a^	0.13 ± 0.07 ^a^
Isoamyl acetate	0.48 ± 0.18 ^a^	2.69 ± 0.36 ^b^	2.47 ± 0.46 ^b^	2.22 ± 0.5 ^b^	2.16 ± 0.37 ^b^
*Aldehydes*					
Furfural	0.00 ± 0.01 ^a^	0.01 ± 0.01 ^a^	0.01 ± 0.02 ^a^	0.01 ± 0.01 ^a^	0.01 ± 0.01 ^a^
*Terpenes and C-13 norisoprenoids*					
*p*-cymene	0.02 ± 0.00 ^a^	0.02 ± 0.00 ^a^	0.02 ± 0.00 ^a^	0.02 ± 0.00 ^a^	0.02 ± 0.00 ^a^
β-ionone	0.01 ± 0.00 ^a^	0.01 ± 0.00 ^a^	0.01 ± 0.00 ^a^	0.01 ± 0.00 ^a^	0.01 ± 0.00 ^a^
